# Cardiovascular Events Risk in Office-Masked Nocturnal Hypertension Defined by Home Blood Pressure Monitoring

**DOI:** 10.1016/j.jacadv.2024.101352

**Published:** 2024-11-07

**Authors:** Takeshi Fujiwara, Satoshi Hoshide, James P. Sheppard, Richard J. McManus, Kazuomi Kario

**Affiliations:** a Division of Cardiovascular Medicine, Department of Medicine, Jichi Medical University School of Medicine, Shimotsuke, Japan; b Nuffield Department of Primary Care Health Sciences, University of Oxford, Oxford, United Kingdom

**Keywords:** cardiovascular disease risk, home blood pressure monitoring, nocturnal blood pressure, office-masked nocturnal hypertension

## Abstract

**Background:**

Nocturnal home blood pressure monitoring (HBPM) may identify people at higher cardiovascular disease (CVD) risk than expected.

**Objectives:**

The aim of this study was to examine the association between office-masked nocturnal hypertension, defined by HBPM, and CVD risk in a clinical practice-based population.

**Methods:**

Prospective observational study including Japanese high cardiovascular-risk participants. Three office blood pressures (OBPs) were taken on two different occasions. Nocturnal home blood pressure (HBP) was measured three times per night for 2 weeks. The association between office-masked nocturnal hypertension and time to first CVD events (fatal and nonfatal stroke or coronary heart disease) was examined using Cox regression.

**Results:**

The cohort included 2,545 participants who were followed for a median of 7.8 years (18,116 person-years), during which 152 CVD events occurred. The proportions of participants with nocturnal normotension (OBP <140/90 mm Hg and nocturnal HBP <120/70 mm Hg), white-coat nocturnal hypertension (OBP ≥140/90 mm Hg and nocturnal HBP <120/70 mm Hg), office-masked nocturnal hypertension (OBP <140/90 mm Hg and nocturnal HBP ≥120/70 mm Hg), and sustained nocturnal hypertension (OBP ≥140/90 mm Hg and nocturnal HBP ≥120/70 mm Hg) were 25.3%, 14.4%, 23.2%, and 37.1%, respectively. Relative to nocturnal normotension, those with both office-masked nocturnal hypertension (adjusted HR: 1.72; 95% CI: 1.01-2.92) and sustained nocturnal hypertension (adjusted HR: 1.75; 95% CI: 1.03-2.96) had similarly increased CVD risk, even after adjustment for daytime HBP values.

**Conclusions:**

Screening for office-masked nocturnal hypertension with HBPM identifies a potentially important group of patients with increased risk for incident CVD events for whom additional preventative measures may be appropriate.

Nocturnal blood pressure (BP) has been increasingly recognized as an important risk factor for hypertension-mediated organ damage and cardiovascular disease (CVD) mortality in hypertensive patients.^1^, ^2^, ^3^, ^4^, ^5^ While 60% of hypertensive patients are considered to have controlled BP on office measurement, around 49% remain with uncontrolled nocturnal hypertension.^6^ Identifying such individuals may enable the optimization of strategies to prevent CVD events in clinical settings. Ambulatory BP monitoring (ABPM) is the standard method to measure nocturnal BP. However, in recent years, we have developed automated home BP monitoring (HBPM) devices for the measurement of nocturnal BP,^7^ and we have shown that nocturnal hypertension defined by HBPM better identifies those at risk of future CVD events compared to nocturnal hypertension defined by ABPM.^8^

Individuals with masked hypertension defined by HBPM are at risk of hypertension-mediated organ damage and have a 2-fold higher risk of CVD compared with those who are normotensive both in and out of the office.^9^, ^10^, ^11^, ^12^ Conventional masked hypertension comprises various subtypes, such as elevated morning BP, daytime BP, and nocturnal BP. Nevertheless, there has been no study to date evaluating CVD prognosis among subtypes of masked hypertension defined by HBPM.

Most hypertension guidelines have recommended that the diagnosis and management of hypertension should be made based on both patients’ office BP and out-of-office BP levels.^13^, ^14^, ^15^, ^16^ Consideration of both office BP and out-of-office BP levels is crucial in the context of routine clinical practice. Diagnosis of masked hypertension also requires both in-office BP and out-of-office BP levels.^13^, ^14^, ^15^, ^16^ We previously demonstrated that people with masked nocturnal hypertension (normal daytime home BP and elevated nocturnal home BP) were at an increased risk of CVD events but we did not use office BP in the definition.^17^ Therefore, in the present analysis we aimed to: 1) examine the association between office-masked nocturnal hypertension (defined as normal office BP and elevated nocturnal home BP) and CVD risk; and 2) to compare the CVD risk associated with office-masked nocturnal hypertension and office-masked daytime hypertension (defined as normal office BP and elevated daytime home BP) in the same study population. We hypothesized that participants with office-masked nocturnal hypertension would show increased risk for CVD events compared to those with normotension.

## Methods

### Ethics committee approval and informed consent

The Institutional Review Board of Jichi Medical University School of Medicine approved the methods, and all participants provided written informed consent to participate and to have their data published.

The data, analytic methods, and study materials that support the findings of the current study are available from the corresponding author upon reasonable request.

### Study design

Details of the J-HOP (Japan Morning Surge-Home Blood Pressure) Nocturnal Blood Pressure Study rationale, design, and procedures have been published previously (see the Supplement).^2^ Briefly, 4,310 outpatients with prior history of or risk factors for CVD were enrolled in the J-HOP study between 2005 and 2012 from primary care practices or university hospital outpatients. The CVD risk factors included hypertension (defined as an office systolic BP [SBP] of ≥140 mm Hg and/or office diastolic BP [DBP] of ≥90 mm Hg, or current use of antihypertensive medication), diabetes or impaired glucose regulation, dyslipidemia, current smoking, chronic kidney disease (CKD), atrial fibrillation, metabolic syndrome, chronic obstructive pulmonary disease, and sleep apnea syndrome (SAS). Any history of CVD events, including angina pectoris, myocardial infarction, and stroke, and the CVD risk factors were ascertained at baseline. Participants with a recent history of CVD events (within 6 months), current hemodialysis treatment, chronic inflammatory disease, or malignancy were excluded. The details of the definitions are shown in the Supplement. Of 4,310 participants, 2,562 participants (59.4% of the total J-HOP sample) performed nocturnal home BP measurements for at least one night within the 14-day study period.^17^

### BP measurements

Details of the methods are described in the Supplemental Appendix. Three office BP readings were taken at 15-second intervals on two different occasions by physicians or nurses using an automatic oscillometric device (HEM-5001, Omron Healthcare) (Supplemental Figure 1), and the mean of the 6 readings was defined as the office BP level. The Omron HEM-5001 BP device uses the same BP measurement algorithm as the validated HEM-737 BP device.^18^

Daytime self-measured home BP measurements were obtained according to the Japanese BP guideline,^19^ using the same validated monitor as used for office BP measurement (HEM-5001, Omron Healthcare). Three home BP readings were taken at 15-second intervals with participants in a seated position in both the morning (within 1 hour of waking and before taking antihypertensive medication) and evening (before going to bed) for 14 consecutive days. For analysis, the first day’s home BP measurements were excluded, and the mean of the remaining morning and evening BP measures was calculated separately. To avoid reporting bias, BP data were automatically stored in the memory of the device and were downloaded to a computer by a physician or nurse during subsequent office visits. For further details of daytime home BP measurements, see the BP measurements in the Supplemental Appendix and Supplemental Figure 2.

Nocturnal home BP was automatically obtained using the same device (HEM-5001, Omron Healthcare): participants wrapped the cuff around the upper arm and pressed a button to start a timer upon going to bed. The device was preset to take three BP measurements at fixed times: 2:00, 3:00, and 4:00 a.m. (one measurement at each time point for a total of three readings per day). Detailed information about the use of the nocturnal HBPM device is provided in Supplemental Figure 2. Nocturnal home BP was defined as the mean of the three nighttime BPs measured in a 14-day study period.

### Definition of BP phenotype

We set the cut-off thresholds for office and home BP based on hypertension guidelines.^14^^,^^15^ The daytime home BP values were defined as the mean of morning and evening home BP values. Initially, office-masked nocturnal hypertension was defined as normotensive office BP (office SBP <140 mm Hg and office DBP <90 mm Hg) with hypertensive nocturnal home BP (nocturnal home SBP ≥120 mm Hg and/or nocturnal home DBP ≥70 mm Hg); white-coat nocturnal hypertension as hypertensive office BP (office SBP ≥140 mm Hg and/or office DBP ≥90 mm Hg) with normotensive nocturnal home BP (nocturnal home SBP <120 mm Hg and nocturnal home DBP <70 mm Hg); sustained nocturnal hypertension as hypertensive office and nocturnal home BPs; and nocturnal normotension as normotensive office and nocturnal home BPs.

In addition, office BP and daytime home BP were used to define BP phenotypes, with office-masked daytime hypertension defined as normotensive office BP with hypertensive daytime home BP (daytime home SBP ≥135 mm Hg and/or daytime home DBP ≥85 mm Hg); white-coat daytime hypertension as hypertensive office BP with normotensive daytime home BP (daytime home SBP <135 mm Hg and daytime home DBP <85 mm Hg); sustained daytime hypertension as hypertensive office and daytime home BPs; and daytime normotension as normotensive office and daytime home BPs.

### Ascertainment of outcomes

Incident total CVD included: 1) fatal and nonfatal stroke, defined as sudden onset of a neurological deficit persisting for ≥24 hours in the absence of any other disease that could account for the symptoms; and 2) fatal and nonfatal coronary heart disease, defined as acute myocardial infarction, angina pectoris requiring percutaneous coronary intervention, and sudden death within 24 hours of the abrupt onset of symptoms. If events occurred on ≥2 occasions, the first occurrence only was included in the analysis.

Each participant’s vital status was ascertained through May 2018. Total CVD events were ascertained by ongoing reports from a general physician at each institute. When participants failed to come to the hospital, we interviewed them or their families by telephone. Additional details are given in the Supplement.

### Statistical analyses

Descriptive statistics are presented as the mean ± SD (for continuous variables) or percentage (for categorical variables). Demographic variables and clinical characteristics were compared across the 4 BP groups (the nocturnal normotensive group; the office-masked nocturnal hypertensive group; the white-coat nocturnal hypertensive group; and the sustained nocturnal hypertensive group) using 1-way analysis of variance (for continuous variables) or chi-square test (for categorical variables). We used the Holm correction for multiple comparisons.^20^

Participants were followed until the first CVD event or otherwise censored at the last study visit. Cumulative incidence estimates were used to compare raw (unadjusted) and adjusted CVD events among BP phenotypes using Gray’s test and Fine-Gray regression, respectively, to account for the competing risk of all-cause death. Adjusted factors included age, gender, body mass index, smoking status, alcohol intake, prevalence of diabetes, prevalence of dyslipidemia, prevalence of CKD, history of CVD (including angina pectoris, myocardial infarction, or stroke), antihypertensive medication use, number of antihypertensive medications, and either: 1) daytime home BP (daytime home SBP and daytime home DBP) when we used nocturnal home BP to define BP phenotypes; or 2) nocturnal home BP (nocturnal home SBP and nocturnal home DBP) when we used daytime home BP to define BP phenotypes.

Cox proportional hazards models were used to calculate HRs and 95% CIs for the likelihood of experiencing a CVD event among BP phenotypes. Schoenfeld residuals were used to confirm the proportional-hazards assumption. HRs were calculated in an unadjusted model (Model 1). Model 2 included adjustment for the covariates mentioned above, and Model 3 included Model 2 components and daytime home BP or nocturnal home BP. These covariates were selected a priori because they have been reported to show correlations with both BP^21^ and CVD risk^22^ and could potentially confound the association between BP and CVD risk.

We conducted a subgroup analysis using only the data of participants taking antihypertensive medications. All statistical analyses were performed with R software, version 4.3.2 (The R Foundation for Statistical Computing). Two-sided *P* values <0.05 were defined as statistically significant.

## Results

### Characteristics of study participants

Among the 2,562 participants, we excluded 17 participants who were lost to follow-up, leaving a sample of 2,545 participants for analysis (Supplemental Figure 3). Table 1 shows baseline characteristics of the included participants for the present analysis in the J-HOP Nocturnal BP study. The mean age was 63.3 ± 10.3 years, 49.0% were male, and 82.6% were using antihypertensive medications.

**Table 1:** Baseline Characteristics of BP Phenotypes Defined Using Office BP and Nocturnal Home BP

	Overall (N = 2,545)	Nocturnal Normotension (n = 645)	White-Coat Nocturnal Hypertension (n = 367)	Office-Masked Nocturnal Hypertension (n = 590)	Sustained Nocturnal Hypertension (n = 943)	*P* Value
Descriptive variables						
Age, y	63.3 ± 10.3	62.4 ± 9.8	64.3 ± 10.1^a^	63.3 ± 9.7	63.5 ± 11.1	0.031
Male	1,246 (49.0)	288 (44.7)	162 (44.1)	296 (50.2)	500 (53.0)^a^^,^^b^	0.002
Body mass index, kg/m^2^	24.3 ± 3.5	23.9 ± 3.6	24.0 ± 3.3	24.4 ± 3.3^a^	24.7 ± 3.5^a^^,^^b^	<0.001
Current smoker	300 (11.8)	85 (13.2)	41 (11.2)	55 (9.3)	119 (12.6)	0.14
Daily drinker	726 (28.5)	166 (25.7)	121 (33.0)	155 (26.3)	284 (30.1)	0.034
Diabetes mellitus	640 (25.1)	137 (21.2)	69 (18.8)	179 (30.3)^a^^,^^b^	255 (27.0)^a^^,^^b^	<0.001
Chronic kidney disease	571 (22.5)	130 (20.2)	66 (18.0)	124 (21.1)	251 (26.7)^a^^,^^b^	0.001
Atrial fibrillation	80 (3.1)	25 (3.9)	9 (2.5)	12 (2.0)	34 (3.6)	0.19
Sleep apnea syndrome	107 (4.2)	30 (4.7)	12 (3.3)	18 (3.1)	47 (5.0)	0.21
Statin use	605 (23.8)	184 (28.5)	80 (21.8)	147 (24.9)	194 (20.6)^a^	0.002
History of CVD	331 (13.0)	80 (12.4)	48 (13.1)	72 (12.2)	131 (13.9)	0.75
Fasting glucose, mg/dL	107.5 ± 27.1	103.9 ± 20.6	105.7 ± 22.3	109.4 ± 30.4^a^	109.5 ± 30.2^a^	<0.001
Total cholesterol, mg/dL	204.3 ± 32.1	204.3 ± 31.2	208.0 ± 34.8	203.5 ± 32.5	203.5 ± 31.3	0.12
High-density lipoprotein cholesterol, mg/dL	58.3 ± 15.6	58.7 ± 15.7	62.1 ± 17.3^a^	56.7 ± 14.6^†^	57.4 ± 15.2^b^	<0.001
Antihypertensive medication use	2,101 (82.6)	512 (79.4)	319 (86.9)^a^	489 (82.9)	781 (82.8)	0.024
Evening administration	736 (28.9)	202 (31.3)	108 (29.4)	156 (26.4)	270 (28.6)	0.30
Number of antihypertensive drugs	1.7 ± 1.2	1.7 ± 1.2	1.8 ± 1.1	1.7 ± 1.2	1.6 ± 1.2	0.34
Calcium channel blockers	1,306 (51.3)	300 (46.5)	199 (54.2)	299 (50.7)	508 (53.9)^a^	0.021
Angiotensin-converting enzyme inhibitors	163 (6.4)	35 (5.4)	25 (6.8)	41 (6.9)	62 (6.6)	0.69
Angiotensin receptor blockers	1,318 (51.8)	340 (52.7)	194 (52.9)	306 (51.9)	478 (50.7)	0.84
Diuretics	732 (28.8)	213 (33.0)	115 (31.3)	178 (30.2)	226 (24.0)^a^^,^^b^^,^^c^	<0.001
β-Blockers	391 (15.4)	101 (15.7)	69 (18.8)	86 (14.6)	135 (14.3)	0.22
α-Blockers	129 (5.1)	25 (3.9)	11 (3.0)	35 (5.9)	58 (6.2)	0.039
BP measures, mm Hg						
Office SBP	140.0 ± 15.4	126.7 ± 9.2	150.4 ± 10.0^a^	129.3 ± 7.6^a^^,^^b^	151.8 ± 11.4^a^^,^^b^^,^^c^	<0.001
Office DBP	81.8 ± 10.3	75.8 ± 7.5	84.7 ± 9.1^a^	77.6 ± 7.7^a^^,^^b^	87.4 ± 10.3^a^^,^^b^^,^^c^	<0.001
Morning home SBP	136.5 ± 14.7	126.4 ± 11.1	133.9 ± 11.5^a^	136.0 ± 12.8^a^^,^^b^	144.6 ± 14.5^a^^,^^b^^,^^c^	<0.001
Morning home DBP	79.3 ± 9.6	74.3 ± 7.6	76.6 ± 8.2^a^	80.1 ± 8.5^a^^,^^b^	83.3 ± 10.0^a^^,^^b^^,^^c^	<0.001
Evening home SBP	128.9 ± 14.3	120.2 ± 11.1	126.1 ± 12.1^a^	129.0 ± 12.9^a^^,^^b^	135.9 ± 14.2^a^^,^^b^^,^^c^	<0.001
Evening home DBP	72.9 ± 9.3	68.9 ± 7.7	70.4 ± 8.3^a^	73.7 ± 8.4^a^^,^^b^	76.1 ± 10.0^a^^,^^b^^,^^c^	<0.001
Daytime home SBP	132.7 ± 13.4	123.3 ± 9.9	130.0 ± 10.5^a^	132.5 ± 11.7^a^^,^^b^	140.3 ± 13.0^a^^,^^b^^,^^c^	<0.001
Daytime home DBP	76.1 ± 8.9	71.6 ± 7.0	73.5 ± 7.6^a^	76.9 ± 7.9^a^^,^^b^	79.7 ± 9.4^a^^,^^b^^,^^c^	<0.001
Nocturnal home SBP	121.1 ± 14.6	107.7 ± 7.0	109.8 ± 6.9^a^	126.4 ± 10.5^a^^,^^b^	131.4 ± 12.6^a^^,^^b^^,^^c^	<0.001
Nocturnal home DBP	69.9 ± 8.7	62.9 ± 4.7	62.6 ± 5.3	73.3 ± 6.4^a^^,^^b^	75.1 ± 8.2^a^^,^^b^^,^^c^	<0.001

Values are mean ± SD or n (%). To compare characteristics among the groups, we used ANOVA with the Holm correction for multiple comparisons. The daytime home BP values were defined as the average of morning and evening home BP values. Statistical significance was defined as *P* < 0.05.

ANOVA = analysis of variance; BP = blood pressure; CVD = cardiovascular disease; DBP = diastolic blood pressure; SBP = systolic blood pressure.

a *P* < 0.05 vs nocturnal normotension group.

b *P* < 0.05 vs white-coat nocturnal hypertension group.

c *P* < 0.05 vs office-masked nocturnal hypertension group.

### Quality control of the home BP measurements

The median (5th-95th percentile interval) number of readings averaged to estimate the morning, evening, nocturnal home BPs were 39 (21-39), 36 (18-39), and 12 (3-39), respectively. In nocturnal home BP measurements, the percentages of participants whose BP measurements were performed three times consecutively on the same night with the differences between each value being <5 mm Hg^14^ were 7.2% in SBP and 16.6% in DBP, respectively. In daytime home BP measurements, the percentages of participants whose BP measurements were performed three times consecutively at each time of morning or evening with the differences between each value being <5 mm Hg^14^ were 22.9% in morning SBP, 53.7% in morning DBP, 26.4% in evening SBP, and 62.3% in evening DBP, respectively. Detailed information on the quality of the home BP measurements is provided in Supplemental Table 1.

### Prevalence of BP phenotypes

**Figure 1:**
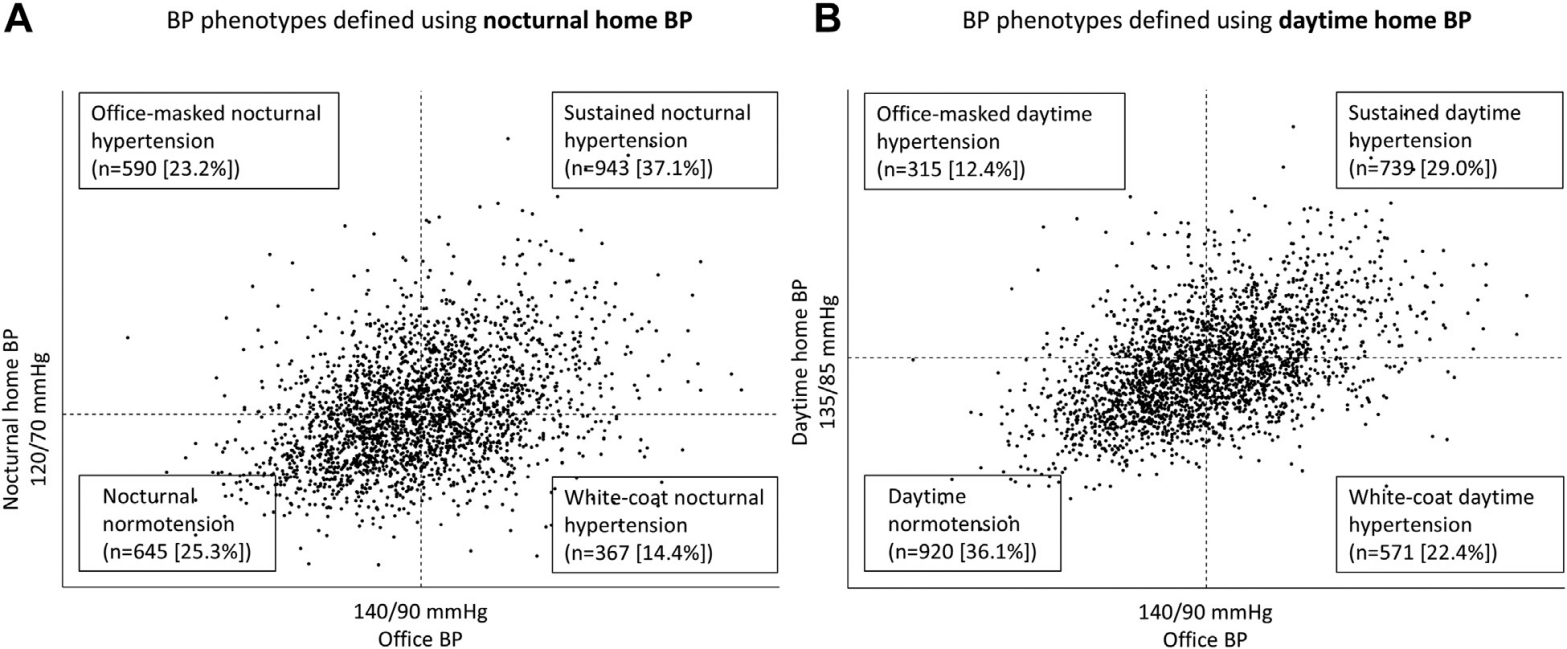
**The Prevalence Rate of Each Blood Pressure Phenotype** Office blood pressure (BP) and home BP levels for each participant were plotted as circles.

The prevalence of office-masked nocturnal hypertension was 23.2% among the overall population and 47.8% among participants with normotensive office BP (Figure 1A). The number of participants who had normotensive office BP and both hypertensive nocturnal and daytime home BP was 229 (9.0% of the total population). Demographic variables and clinical characteristics of BP phenotypes defined using daytime home BPs are shown in Supplemental Table 2. Nocturnal home BP values were similar between participants with office-masked nocturnal hypertension and those with office-masked daytime hypertension (126.4/73.3 mm Hg vs 125.6/71.2 mm Hg), but daytime home BP was lower in participants with office-masked nocturnal hypertension compared with those with office-masked daytime hypertension (132.5/76.9 mm Hg vs 142.6/80.0 mm Hg) (Table 1, Supplemental Table 2).

### Associations between each BP phenotype and CVD outcomes

**Figure 2:**
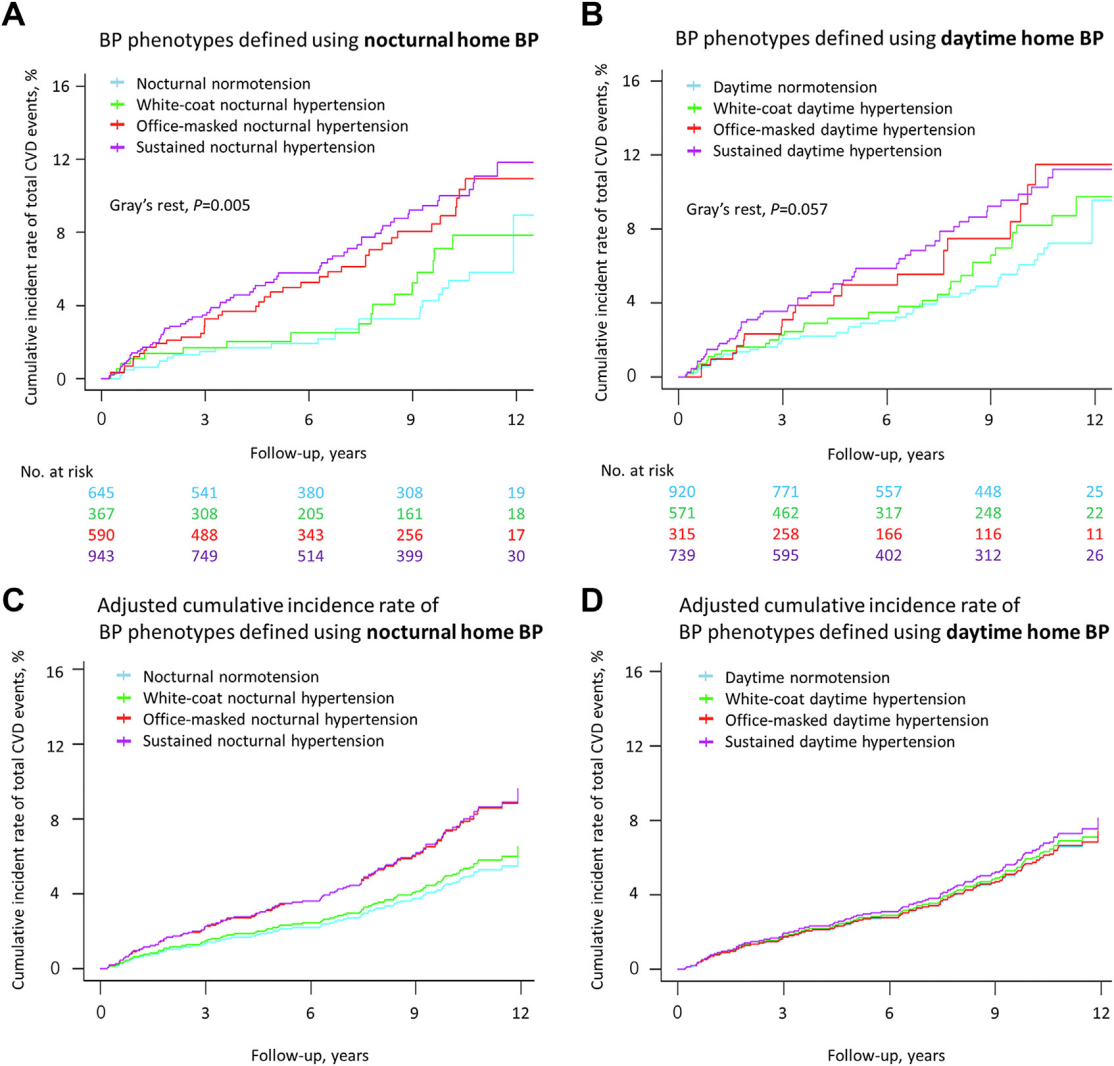
**Cumulative Incidence of Cardiovascular Disease Events by the 4 Blood Pressure Groups** Cumulative incidence of total cardiovascular disease (CVD) events by the four blood pressure (BP) groups is shown. (A, B) The unadjusted and (C, D) adjusted cumulative incidence rates were calculated using Gray’s test and the Fine-Gray model, respectively, accounting for the competing risk of death. Analyses were performed for both (C) office BP with daytime home BP and (D) office BP with nocturnal home BP.

During a median (25th-75th percentiles) follow-up of 7.8 (3.9-11.0) years (18,116 person-years), 152 total CVD events (8.4/1,000 person-years) occurred. When BP phenotypes were defined using nocturnal home BP, the office-masked nocturnal hypertensive and sustained nocturnal hypertensive groups had higher incidence rates of total CVD events compared with the nocturnal normotensive group, even after adjustment for covariates including daytime home BP levels (Figures 2A and 2C). When BP phenotypes were defined using daytime home BP, the office-masked daytime hypertensive group and sustained daytime hypertensive group tended to have higher incidence rates of total CVD events compared with the daytime normotensive group, but the difference was not statistically significant (Figures 2B and 2D).

With adjustments for covariates, results from the Cox models suggested that both the office-masked nocturnal hypertensive and sustained nocturnal hypertensive groups had greater risk for a CVD event compared with the nocturnal normotensive group when BP phenotypes were defined using nocturnal home BP: office-masked nocturnal hypertension (adjusted HR: 1.80; 95% CI: 1.08-3.01); sustained nocturnal hypertension (adjusted HR: 1.96; 95% CI: 1.21-3.15) (Model 2 in Table 2). When we further adjusted for daytime home BP levels, the results were similar: office-masked nocturnal hypertension (adjusted HR: 1.72; 95% CI: 1.01-2.92); sustained nocturnal hypertension (adjusted HR: 1.75; 95% CI: 1.03-2.96) (Model 3 in Table 2). When BP phenotypes were defined using daytime home BP, the sustained daytime hypertensive group had greater risk for total CVD events compared with the daytime normotensive group in the unadjusted model (Model 1 in Table 3); however, this was not the case after adjustment for covariates (Model 2 and Model 3 in Table 3). Neither white-coat nocturnal hypertension nor white-coat daytime hypertension was associated with increased risk of total CVD events (Central Illustration).

**Table 2:** Cardiovascular Event Risk for BP Phenotypes Defined by Home BP Monitoring: BP Phenotypes Defined Using Office BP and Nocturnal Home BP

	Nocturnal Normotension (n = 645)	White-Coat Nocturnal Hypertension (n = 367)	Office-Masked Nocturnal Hypertension (n = 590)	Sustained Nocturnal Hypertension (n = 943)
Number of events (per 1,000 person-years; 95% CIs)	24 (5.2; 3.5-7.8)	17 (6.5; 4.1-10.4)	41 (9.8; 7.2-13.3)	70 (10.4; 8.3-13.2)
Model 1 (unadjusted)	1.00 (reference)	1.28 (0.69-2.38)	1.94 (1.17-3.21)	2.14 (1.35-3.40)
Model 2 (adjusted)	1.00 (reference)	1.22 (0.65-2.29)	1.80 (1.08-3.01)	1.96 (1.21-3.15)
Model 3 (Model 2 + daytime home BP)	1.00 (reference)	1.15 (0.61-2.17)	1.72 (1.01-2.92)	1.75 (1.03-2.96)

**Table 3:** Cardiovascular Event Risk for BP Phenotypes Defined by Home BP Monitoring: BP Phenotypes Defined Using Office BP and Daytime Home BP

	Daytime Normotension (n = 920)	White-Coat Daytime Hypertension (n = 571)	Office-Masked Daytime Hypertension (n = 315)	Sustained Daytime Hypertension (n = 739)
Number of events (per 1,000 person-years; 95% CIs)	44 (6.7; 5.0-9.0)	32 (7.9; 5.6-11.1)	21 (9.4; 6.1-14.3)	55 (10.5; 8.0-13.6)
Model 1 (unadjusted)	1.00 (reference)	1.24 (0.78-1.95)	1.57 (0.93-2.64)	1.69 (1.13-2.51)
Model 2 (adjusted)	1.00 (reference)	1.15 (0.73-1.83)	1.24 (0.73-2.12)	1.47 (0.98-2.22)
Model 3 (Model 2 + nocturnal home BP)	1.00 (reference)	1.08 (0.68-1.72)	1.02 (0.59-1.78)	1.12 (0.70-1.77)

The adjusted HR (95% CIs) associated with each BP group is shown. The daytime home BP values were defined as the average of morning and evening home BP values. Adjusted factors for Model 2 included age, gender, body mass index, smoking status, alcohol intake, prevalence of diabetes, prevalence of dyslipidemia, prevalence of chronic kidney disease, history of cardiovascular disease (including angina pectoris, myocardial infarction, or stroke), antihypertensive medication use, and number of antihypertensive medications. Adjustment factors for Model 3 included Model 2 components and daytime home BP (daytime home SBP + daytime home DBP) or nocturnal home BP (nocturnal home SBP + nocturnal home DBP).

BP = blood pressure; DBP = diastolic blood pressure; HBP = home blood pressure; SBP = systolic blood pressure.

**Central Illustration:**
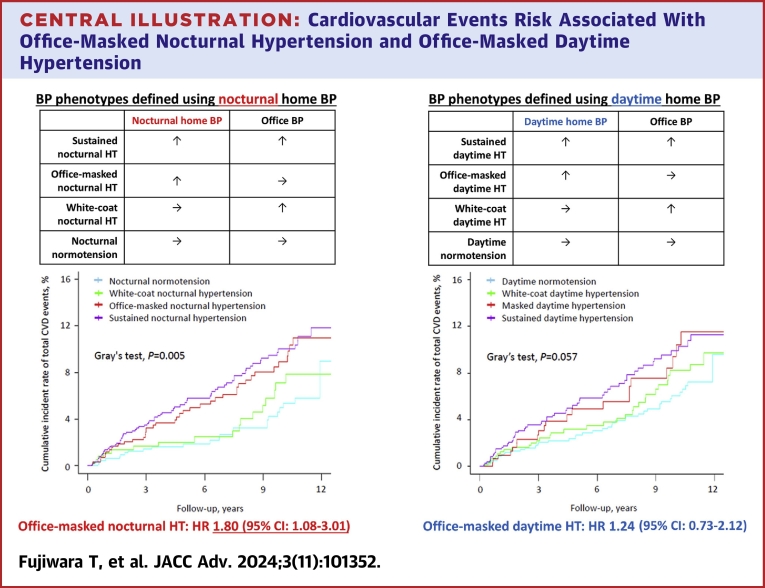
**Cardiovascular Events Risk Associated With Office-Masked Nocturnal Hypertension and Office-Masked Daytime Hypertension** BP = blood pressure; CVD = cardiovascular disease; HT = hypertension.

When we used the data of only those participants taking antihypertensive medications, the results were similar (Supplemental Table 3).

## Discussion

### Main study findings

In this nationwide practice-based observational study of 2,545 Japanese with a prior history of or risk factors for CVD, we found that office-masked nocturnal hypertension defined by HBPM (vs normotensive office and nocturnal home BPs) was associated with increased risk for a CVD event, independently of daytime home BP levels. The magnitude of CVD risk in the office-masked nocturnal hypertensive group was similar to that in the sustained nocturnal hypertensive groups. Despite the 10 mm Hg-lower daytime SBP and 0.8 mm Hg-higher nocturnal home SBP in the office-masked nocturnal hypertension group compared to the office-masked daytime hypertension group, the office-masked nocturnal hypertension group exhibited a higher CVD risk; office-masked daytime hypertension was not associated with CVD risk. White-coat hypertension yielded no association with CVD risk. This is the first study to demonstrate the total CVD risk associated with office-masked nocturnal hypertension and to compare the CVD risk between office-masked nocturnal hypertension and office-masked daytime hypertension in the same study population. Our findings suggest that the assessment of nocturnal home BP levels is important to improve risk stratification of CVD in clinical practice.

### Comparison with previous literature

In the J-HOP Nocturnal BP Study, participants with office-masked nocturnal hypertension had a 1.5- to 2.0-fold higher risk of CVD events compared to those with nocturnal normotension. This result was consistent with our previous results obtained using only HBPM: participants with masked nocturnal hypertension (normal daytime home BP and elevated nocturnal home BP) (adjusted HR: 1.57; 95% CI: 1.00-2.46) and those with sustained hypertension (both elevated daytime and nocturnal home BPs) (adjusted HR: 1.97; 95% CI: 1.26-3.06) were shown to have increased CVD risk in our previous study.^17^ Those results were in line with other previous reports that found home BP level to be a stronger predictor for CVD events than office BP levels.^23^^,^^24^ Furthermore, the present results indicate that nocturnal home BP contributes more to CVD risk than daytime home BP. The IDACO (International Database on Ambulatory Blood Pressure in Relation to Cardiovascular Outcome), a population-based cohort study (n = 11,135 and 20.3% received antihypertensive medication), demonstrated that nighttime SBP was significantly associated with an increased risk of total mortality even after adjustment for 24-hour SBP levels, although in that study, the significance of daytime SBP disappeared after adjustment for the nighttime SBP level.^3^ Similar associations were observed in the Spanish Ambulatory Blood Pressure Registry, a large population-based cohort study (n = 59,124, and 59.4% received antihypertensive medication).^5^ That study found that nighttime SBP was associated with an increased risk of all-cause and cardiovascular death even after adjustment for daytime SBP levels; on the other hand, the significant risks of all-cause and cardiovascular death of daytime SBP disappeared after adjustment for nighttime SBP level.^5^ In the present analysis, we extended these findings by demonstrating that office-masked nocturnal hypertension defined by HBPM conferred a higher risk for CVD events compared with nocturnal normotension, even after adjustment for daytime home BP levels. This result highlights the potential of evaluating nocturnal BP levels using home monitoring along with conventional daytime home BP levels in clinical practice for accurate stratification of CVD risk.

There are several possible explanations for our finding that office-masked nocturnal hypertension and sustained nocturnal hypertension were associated with increased CVD risk even after adjusting for daytime home BP levels. Sympathetic activity is reduced during the nighttime compared to the daytime since there is little external stimulation during sleep. However, sympathetic tone during sleep can be elevated, especially in patients with diabetes^25^ or SAS,^26^ and elevated sympathetic tone causes an abnormal nocturnal BP increase. In patients with CKD, the elevation in circulating volumes of blood and interstitial fluid, especially when in a supine position during sleep, contributes to an increase of nocturnal BP levels.^27^ However, in the present study, modeling adjustments for the prevalence of diabetes and CKD in the association between office-masked nocturnal hypertension and sustained nocturnal hypertension and their CVD risk revealed that these associations remained statistically significant. It is yet to be clearly elucidated whether these factors may contribute to increased CVD risk through the elevation of nocturnal BP levels.

Another possible explanation concerns the use of antihypertensive medications. In this study population, 82.6% of participants were taking antihypertensive medications. Physicians often adjust antihypertensive medications based on office BP or conventional daytime home BP levels.^13^, ^14^, ^15^, ^16^ In fact, daytime home BP levels in participants with office-masked nocturnal hypertension were largely controlled (Table 1). The results of this study might represent residual risks in individuals with office-masked nocturnal hypertension and sustained nocturnal hypertension that would remain unnoticed without measurement of nighttime BP levels. Other factors, such as arterial stiffness, impaired endothelial function, seasonal BP variation, adherence to antihypertensive medication, insomnia, or psychosocial stress, could be related to the high CVD risks of office-masked nocturnal hypertension and sustained nocturnal hypertension.^28^, ^29^, ^30^, ^31^, ^32^, ^33^ Further investigation will be required to elucidate the pathophysiological mechanisms underlying elevations of nocturnal BP relative to daytime BP and the specific risks conferred by this disparity.

Our present results indicated that about a quarter of participants in this clinical practice population had office-masked nocturnal hypertension. In a cross-sectional study conducted in a tertiary hospital in Buenos Aires (917 hypertensive participants, mean age 65.1 ± 14.7 years, 43.3% male, 73.9% participants used antihypertensive mediations), the prevalence of masked nocturnal hypertension (defined as normotensive office and daytime ambulatory BP, but elevated nocturnal ambulatory BP) was 9.7%.^34^ In the cross-sectional analysis of the HI-JAMP (Home-Activity ICT-based Japan Ambulatory Blood Pressure Monitoring Prospective) study, which is a general-practitioner-based, nationwide, multicenter prospective study (2,322 hypertensive participants with antihypertensive medication use, mean age 69.2 ± 11.5 years, 53.2% male), the prevalence of masked uncontrolled nocturnal hypertension (defined as normotensive office BP but elevated nocturnal ambulatory BP) was 13.8%.^35^ Differences in the definition of masked nocturnal hypertension or the characteristics or ethnicities of the study participants may have contributed to these differences in prevalence of masked nocturnal hypertension, but HBPM would have the potential to identify a population with high CVD risk of office-masked nocturnal hypertension more effectively than ABPM.^6^ Although ABPM has historically been the gold standard for the measurement of nocturnal BP levels, it has low tolerability due to sleep disturbance and impact on daily activities.^36^ On the other hand, previous studies have reported that nocturnal BP measurement using HBPM is preferable to that using ABPM^37^^,^^38^ due to its easy availability and low cost.^14^ In addition, multiple values of nocturnal home BP can be obtained over time and are reproducible.^39^ Furthermore, we previously demonstrated that nocturnal BP measured by HBPM was more strongly associated with hypertension-mediated organ damage than that measured by ABPM, and that nocturnal hypertension defined by HBPM was associated with increased risk of CVD, whereas that defined by ABPM showed no such association.^8^^,^^40^ Further studies are needed to assess the effectiveness on CVD outcomes of an HBPM-based treatment strategy, including the management of nocturnal BP levels.

### Study strengths and limitations

The strengths of this study include its nationwide scope, the use of the same device for measuring office BP and home BP (both daytime and nocturnal), and the high participant retention rate. However, there are some limitations. First, 82.6% of our recruited participants were on antihypertensive medication, and data on medication changes and associated BP changes were not available during the follow-up period. Second, the number of study participants could have influenced the statistical power to evaluate the CVD risk among participants with office-masked daytime hypertension, since previous studies have consistently indicated an increased risk of CVD in individuals with office-masked daytime hypertension defined using HBPM.^9^, ^10^, ^11^, ^12^ Third, our findings were obtained from a high CVD risk population and cannot be generalized to broader populations. Fourth, since this was an observational study, we were unable to determine causality in the findings. Fifth, given the well-established relationship between SAS and elevated nocturnal BP,^41^ the potential underdiagnosis of SAS might impact the applicability of our findings. Finally, our findings may not be generalized to other ethnic groups.

## Conclusions

In this Japanese practice-based observational study, we provide the first direct evidence of increased CVD risk associated with office-masked nocturnal hypertension. Nocturnal home BP measurement may be considered as a complementary strategy for estimating CVD risk in clinical settings, in addition to conventional daytime BP measurements. Further studies are warranted to assess whether reductions in nocturnal home BP can help to prevent CVD events among individuals with office-masked nocturnal hypertension.

Perspectives **COMPETENCY IN MEDICAL KNOWLEDGE:** Office-masked nocturnal hypertension defined by home BP monitoring is associated with increased risk for CVD events, independently of daytime home BP levels.

**COMPETENCY IN PATIENT CARE:** Nocturnal home BP monitoring, along with conventional daytime home BP monitoring, could have potential to identify patients at high risk of CVD in clinical practice.

**TRANSLATIONAL OUTLOOK:** More studies are needed to determine whether antihypertensive treatment of office-masked hypertension improves CVD outcomes.

## Funding support and author disclosures

Dr Fujiwara received funding from the 10.13039/501100008667SENSHIN Medical Research Foundation. Dr Sheppard received funding from the 10.13039/100010269Wellcome Trust/10.13039/501100000288Royal Society via a Sir Henry Dale Fellowship (ref: 211182/Z/18/Z). For the purpose of open access, the author has applied a CC BY public copyright license to any author-accepted manuscript version arising from this submission. Dr McManus is supported by the NIHR Oxford and Thames Valley Applied Research Consortium and is the recipient of an NIHR Senior Investigator Award. Dr Kario is supported by the 21st Century Center of Excellence Project run by Japan’s Ministry of Education, Culture, Sports, Science, and Technology; a grant from the Foundation for Development of the Community (Tochigi, Japan); a grant from 10.13039/100016274Omron Healthcare, Co, Ltd; a Grant-in-Aid for Scientific Research (B) (21390247) from the 10.13039/501100001700Ministry of Education, Culture, Sports, Science and Technology (MEXT) of Japan, 2009 to 2013; and funds from the MEXT-Supported Program for the Strategic Research Foundation at Private Universities, 2011 to 2015 Cooperative Basic and Clinical Research on Circadian Medicine (S1101022). The funding sponsors had no role in designing or conducting this study; in the collection, management, analysis, or interpretation of the data; in the preparation of the article; or in the decision to submit the article for publication. Dr McManus has worked with Omron to develop a UK-based telemonitoring system for which his institution received consultancy and licensing fees. Dr Kario has received research grants from 10.13039/100016274Omron Healthcare and A&D Co. All other authors have reported that they have no relationships relevant to the contents of this paper to disclose.
