# Supplementary material for: Cardiovascular Events Risk in Office-Masked Nocturnal Hypertension Defined by Home Blood Pressure Monitoring
Source: JACC Adv. 2024 Nov 7;3(11):101352. doi: 10.1016/j.jacadv.2024.101352 (PMC11588851; doi:10.1016/j.jacadv.2024.101352)
Supplement: Supplemental material [file mmc1.docx]

**SUPPLEMENTAL APPENDIX**

**Methods**

**J-HOP study**

The Japan Morning Surge-Home Blood Pressure (J-HOP) Study is a prospective observational study evaluating the use of home blood pressure (BP) to predict cardiovascular disease (CVD) events in Japanese individuals with any of the following CVD risk factors: hypertension, impaired glucose regulation (defined as either impaired fasting glucose or impaired glucose tolerance) or diabetes mellitus, dyslipidemia, current smoking (and/or current chronic obstructive pulmonary disease), chronic kidney disease (CKD), atrial fibrillation, metabolic syndrome, and sleep apnea syndrome. The exclusion criteria for the J-HOP Study were a recent history of CVD events (within 6 months), current hemodialysis treatment, chronic inflammatory disease, or malignancy. Diagnostic criteria of the CVD risk factors were hypertension, defined as an office systolic BP (SBP) of ≥140 mmHg and/or office diastolic BP (DBP) of ≥90 mmHg, or current use of antihypertensive medication; impaired fasting glucose, defined as a fasting glucose level of ≥110 mg/dl; impaired glucose tolerance, defined as a glucose level of ≥140 mg/dl at 2 h after a 75 g oral glucose tolerance test; diabetes, defined as self-reported history of a physician’s diagnosis, diabetes medication use, fasting blood glucose level ≥126 mg/dL or non-fasting glucose level ≥200 mg/dL; dyslipidemia, defined as a total cholesterol level of ≥240 mg/dl or treated dyslipidemia; CKD, defined as a serum creatinine-based estimated glomerular filtration rate <60 mL/min/1.73m^2^ and/or microalbuminuria, defined as a urine albumin-to-creatinine ratio ≥30 mg/g·Cr^1^; metabolic syndrome, defined according to the guidelines of the Examination Committee of the Criteria for Metabolic Syndrome in Japan published in April 2005^2^; and sleep apnea syndrome, defined as an apnea-hypopnea index of ≥15 events/h by overnight sleep polysomnography.

In Japan, there are 47 administrative divisions (prefectures). In 25 of the prefectures (Tochigi, Aichi, Yamaguchi, Nagano, Miyazaki, Ibaraki, Hiroshima, Kumamoto, Hyogo, Tottori, Chiba, Saitama, Niigata, Fukushima, Oosaka, Shiga, Gunma, Kanagawa, Tokyo, Toyama, Mie, Yamagata, Gifu, Saga, and Nara), a total of 75 doctors at 71 institutions (45 primary practices, 22 hospital-based outpatient clinics, and 4 specialized university hospitals) agreed with the aims of this study and collected prospective data from individuals who agreed to participate in this project.

**BP measurements**

Office BP was measured by physicians or nurses using an upper-arm cuff oscillometric BP device (HEM-5001; Omron Healthcare, Kyoto, Japan). The Omron HEM-5001 home BP device uses the same BP measurement algorithm as used in the HEM-737 home BP device, which was validated in a previous study.^3^ Three office BP readings were taken at 15-sec intervals in a sitting position.^4^ The analyses were performed using the mean values of six readings obtained during two office visits (before and after the home BP measurements). We advised the patients to take their morning medication as usual on the days when they were visiting the clinic.

Self-measured home BP values were obtained using the same device (HEM-5001). The patients were instructed to place a cuff of appropriate size on the same arm throughout the measurements and to measure their BP in a sitting position after ≥2 min of rest according to the Japanese Society of Hypertension 2004 guidelines.^5^ Three home BP readings were taken at 15-sec intervals in a sitting position in both the morning and evening for 14 days. Morning BP was measured within 1 h of waking, after urination, before breakfast and before taking antihypertensive medication. Evening BP was measured before going to bed, and the patients were instructed to avoid measuring their BP just after taking a bath, drinking alcohol or smoking. The morning and evening BP data were automatically stored in the memory of the BP device and were downloaded to a computer by a physician or nurse during clinic visits. The data were then sent to the study control center (Jichi Medical University, Tochigi, Japan). After exclusion of the data from the first day, the averages of all home BP measures taken three times in the morning (morning home BP) and three times in the evening (evening home BP) for 13 days (78 readings in total) were separately calculated by the study coordinator, who was blinded to the clinical characteristics of the study participants.

**Laboratory and other examinations**

Blood and spot urine samples were collected in the morning in a fasting state at study enrollment. The blood samples were centrifuged at 3000 ×g for 15 min at room temperature. Plasma/serum samples after separation and urine samples were stored at 4°C in refrigerated containers and sent to a commercial laboratory (SRL Inc., Tokyo) within 24 h. Serum samples after separation were also stored at −80°C in a refrigerator. All assays were performed within 24 h of sample collection at a single laboratory center (SRL Inc.). Total cholesterol values were measured using the cholesterol dehydrogenase-UV method. Serum high-density-lipoprotein cholesterol was determined by the direct method using cholesterol oxidase. Questionnaires were used to collect information on demographics, smoking and drinking status, medical history and medication use.

**Outcomes ascertainment**

Outcomes were categorized as follows. (i) Fatal and nonfatal stroke, defined as sudden onset of a neurological deficit persisting for ≥24 h in the absence of any other disease that could account for the symptoms. Stroke events included cerebral infarction, cerebral hemorrhage, and subarachnoid hemorrhage based on the findings of brain computed tomography or magnetic resonance imaging. Transient ischemic brain attacks (i.e., those in which the neurological deficit was completely resolved within 24 h of the onset of symptoms) were not calculated as stroke events. (ii) Fatal and nonfatal coronary heart disease, defined as acute myocardial infarction, angina pectoris requiring percutaneous coronary intervention, and sudden death within 24 h of the abrupt onset of symptoms. Criteria for myocardial infarction included definite electrocardiographic findings (i.e., ST elevation), typical or atypical symptoms together with electrocardiographic findings and abnormal enzymes, or typical symptoms and abnormal cardiac enzymes with or without electrocardiographic findings. If events occurred on ≥2 occasions, the first occurrence was included in the analysis. Evidence on the above CVD outcomes was ascertained by ongoing reports from a general physician at each institute. When participants failed to come to the hospital, we interviewed them or their families, or both, by telephone. The end point committee adjudicated all events by reviewing the participants’ files and source documents or by requesting more detailed written information from investigators. The committee was blinded to individual clinical characteristics including home BP data. A final follow-up survey to reconfirm the clinical outcomes was performed from December 2017 to May 2018.

**References**

1. Japanese Society of Nephrology. Evidence-based Clinical Practice Guideline for CKD 2013. *Clin Exp Nephrol.* 2014;18:346-423.

2. Committee to Evaluate Diagnostic Standard for Metabolic Syndrome. Definition and the Diagnostic standard for metabolic syndrome. *Nippon Naika Gakkai Zasshi.* 2005;94:794-809. (in Japanese).

3. Anwar YA, Giacco S, McCabe EJ, Tendler BE, White WB. Evaluation of the efficacy of the Omron HEM-737 IntelliSense device for use on adults according to the recommendations of the Association for the Advancement of Medical Instrumentation. *Blood Press Monit.* 1998;3:261-265.

4. Yarows SA, Patel K, Brook R. Rapid oscillometric blood pressure measurement compared to conventional oscillometric measurement. *Blood Press Monit.* 2001;6:145-147.

5. Imai Y, Otsuka K, Kawano Y, Shimada K, Hayashi H, Tochikubo O, Miyakawa M, Fukiyama K, Japanese Society of Hypertension. Japanese society of hypertension (JSH) guidelines for self-monitoring of blood pressure at home. *Hypertens Res.* 2003;26:771-782.

**Participants and participating centers**

Kazuomi Kario: Jichi Medical University School of Medicine; Satoshi Hoshide: Jichi Medical University School of Medicine; Hajime Haimoto: Haimoto Clinic; Kayo Yamagiwa: Yamagiwa Clinic; Kiyoshi Uchiba: Oooka Clinic; Syouichirou Nagasaka: Jichi Medical University School of Medicine; Yuichiro Yano: Nango Clinic; Kazuo Eguchi: Jichi Medical University School of Medicine and International University of Health and Welfare Hospital; Yoshio Matsui: Jichi Medical University and Hagi city Mishima Clinic; Motohiro Shimizu: Ogi city Fukukawa Clinic and Heigun Clinic; Akira Nakamura: Chukyo Clinic; Joji Ishikawa: Jichi Medical University School of Medicine and Koga Red Cross Hospital; Shizukiyo Ishikawa: Jichi Medical University School of Medicine and Washiya Hospital; Motoki Fukutomi: Simonoseki city Tsunoshima Clinic; Tomoyuki Kabutoya: Jichi Medical University School of Medicine and Ojikano Central Hospital and Chichibu Municipal Hospital; Kyousei Souda: Souda Clinic; Michiaki Nagai: Syoubara Red Cross Hospital and Syoubara city National Health Insurance Clinic; Seiichi Sibazaki: Ogi city Fukukawa Clinic; Hideyuki Uno: Jichi Medical University School of Medicine and Noda Hospital; Sachiyo Ogata: Joriku-Omiya Saiseikai Hospital; Yoshifumi Nojiri: Joetsu Community Medical Center Hospital; Ryuji Inoue: Kanzaki General Hospital; Kazuhiko Kotani: Tottori University Hospital; Satoshi Yamada: Yamada Clinic; Takeshi Mitsuhashi: Jichi Medical University School of Medicine; Hiroaki Tsukao: Yamashita Clinic; Tetsuya Aoki: Akasaki Clinic; Toshio Kuroda: Kuroda Internal Medicine and Cardiovascular Clinic; Yutaka Nakajima: Shimonoseki city Toyota Central Hospital; Akinori Hirai: Nagahama Red Cross Hospital; Hareaki Yamamoto: Yamamoto Clinic; Tsuneo Oowada: Oowada Internal Medicine and Gastrointestinal Clinic; Masaru Ichida: Jichi Medical University School of Medicine; Setsuko Katou: Katou Clinic of Internal Medicine; Takahiro Komori: Jichi Medical University School of Medicine and Utsunomiya Social Insurance Hospital and Kurai Kiyohiko Memorial Hospital; Sigeki Nishizawa: Nishizawa Clinic of Internal Medicine; Kazuhiro Murata: Ooshima Clinic; Takashi Utsu: Shiga Medical University; Toru Kato: Koyanagi Memorial Hospital; Osamu Kuwasaki: Kuwasaki Clinic of Internal Medicine; Yutaka Shimada: Kyaranoki Care Center; Yoshihiro Yonezawa: Yonezawa Clinic; Eiji Inoue: Inoue Clinic of Internal Medicine; Masatoshi Matsumoto: Jichi Medical University School of Medicine; Toru Kimura: Iiduka Clinic; Kenichi Sakakura: Kumano city Kiwa Clinic; Shingo Shikano: Ibuki Shikano Clinic; Kazuhiro Handa: Handa Clinic; Kouichirou Abe: Abe Clinic of Internal Medicine; Motoyuki Ishiguro: Ishiguro Clinic; Yoshio Onogaki: Onogaki Clinic; Hiroshi Kubo: Hiro Clinic of Cardiovascular Medicine and Gastrointestinal; Kouichi Tokai: Kamihira Clinic; Ryou Touji: Touji Clinic; Akiya Nakamoto: Nakamoto Clinic of Internal Medicine; Youichi Ehara: Yoshii Chuo Clinic; Masahiro Toshima: Kamiichi General Hospital; Nobuyuki Adachi: Adachi Clinic of Internal Medicine; Nobuo Takahashi: Takahashi Family Clinic; Masashi Tanaka: Manba Clinic; Fumihiko Eto: Privcare Family Clinic; Masahisa Shinpo: Jichi Medical University School of Medicine; Katsumi Tanaka: Youga Urban Clinic; Takeshi Takemi: Clinic Jingu-Mae; Masayuki Nagata: Nakata Clinic; Yukihiro Hojo: Jichi Medical University School of Medicine; Yoko Hoshide: Satou Clinic; Fumihiko Yasuma: Suzuka National Hospital; Hajime Yanagisawa: Sudou Hospital; Yukitaka Anraku: Omocyanomachi Internal Medicine Clinic; Shuichi Ueno: Jichi Medical University School of Medicine; Ryousuke Kusaba: Saitama Tsukuba Hospital; Naoshi Suzuki: Washiya Hospital; Nobuyuki Maki: Kamogawa City National Health Insurance Hospital.

(75 physicians and 71 institutes)

| **Supplemental Table 1. Quality control of home BP measurement** | | | | | | | | |
| --- | --- | --- | --- | --- | --- | --- | --- | --- |
| 1. **Number of measurement days per participant** | | | | | | | | |
|  | **Metric** | | | | | | | |
| **Measurement timing** | **Median** | | **P25–P75** | | **P10–P90** | | **P5–95** | |
| Morning | 13 | | 12–13 | | 10–13 | | 6–13 | |
| Evening | 13 | | 12–13 | | 9–13 | | 6–13 | |
| Nighttime | 4 | | 2–11 | | 1–13 | | 1–13 | |
| 1. **Number of BP readings per participant** | | | | | | | | |
|  | **Metric** | | | | | | | |
| **Measurement timing** | **Median** | | **P25–P75** | | **P10–P90** | | **P5–95** | |
| Morning | 39 | | 36–39 | | 30–39 | | 21–39 | |
| Evening | 36 | | 33–39 | | 27–39 | | 18–39 | |
| Nighttime | 12 | | 6–32 | | 3–38 | | 3–39 | |
| 1. **Number of participants with each of the three BP measurements differing by less than 5 mmHg** | | | | | | | | |
|  | **Home BP measurement** | | | | | | | |
| **Days** | **Morning SBP** | **Morning DBP** | | **Evening SBP** | **Evening DBP** | **Nocturnal SBP** | | **Nocturnal DBP** |
| Day 2 | 573 (23.0) | 1,309 (52.6) | | 601 (24.5) | 1,418 (57.8) | 62 (6.0) | | 164 (15.9) |
| Day 3 | 556 (22.6) | 1,297 (52.6) | | 604 (24.8) | 1,513 (62.2) | 77 (7.1) | | 174 (16.1) |
| Day 4 | 554 (22.5) | 1,293 (52.6) | | 626 (25.9) | 1,477 (61.0) | 76 (6.7) | | 185 (16.3) |
| Day 5 | 572 (23.5) | 1,278 (52.5) | | 649 (27.0) | 1,478 (61.4) | 89 (7.7) | | 194 (16.8) |
| Day 6 | 541 (22.3) | 1,319 (54.3) | | 645 (26.7) | 1,505 (62.3) | 85 (7.8) | | 195 (17.9) |
| Day 7 | 533 (22.0) | 1,306 (53.9) | | 628 (26.2) | 1,496 (62.4) | 88 (7.6) | | 197 (16.9) |
| Day 8 | 550 (23.7) | 1,299 (56.1) | | 631 (27.6) | 1,496 (65.4) | 77 (7.1) | | 174 (16.0) |
| Day 9 | 544 (23.5) | 1,262 (54.5) | | 594 (26.1) | 1,444 (63.4) | 83 (7.7) | | 192 (17.8) |
| Day 10 | 529 (23.0) | 1,233 (53.5) | | 651 (28.5) | 1,500 (65.7) | 83 (7.9) | | 168 (16.0) |
| Day 11 | 545 (23.7) | 1,252 (54.6) | | 619 (27.1) | 1,427 (62.6) | 85 (7.8) | | 177 (16.2) |
| Day 12 | 526 (22.9) | 1,280 (55.7) | | 621 (27.3) | 1,432 (63.0) | 78 (7.2) | | 179 (16.6) |
| Day 13 | 508 (22.4) | 1,204 (53.0) | | 585 (26.1) | 1,406 (62.7) | 74 (6.7) | | 179 (16.2) |
| Day 14 | 516 (23.2) | 1,216 (54.7) | | 505 (26.6) | 1,239 (65.3) | 72 (6.6) | | 188 (17.2) |
| Data are expressed as number (percentage). In the case of daytime BP measurement, the percentage is shown based on the number of participants for whom all three consecutive measurements were taken at each time. In the case of nocturnal BP measurements, the percentage is shown based on the number of participants who measured their nocturnal BP three times on the same day. P25-P75 indicates the 25th–75th percentile interval, P10-P90 the10th–90th percentile interval, P5-P95 the 5th–95th percentile interval. BP indicates blood pressure; DBP, diastolic blood pressure; SBP, systolic blood pressure. | | | | | | | | |

| **Supplemental Table 2. Baseline characteristics of BP phenotypes defined using office BP and daytime home BP** | | | | | |
| --- | --- | --- | --- | --- | --- |
|  | **Daytime**  **normotension**  (n=920) | **White-coat**  **daytime**  **hypertension**  (n=571) | **Office-masked daytime**  **hypertension**  (n=315) | **Sustained**  **daytime**  **hypertension**  (n=739) | ***P* value** |
| **Descriptive variables** | | | | | |
| Age, years | 62.1±9.5 | 63.3±10.0 | 64.9±10.3^*^ | 64.1±11.4^*^ | <0.001 |
| Male, n (%) | 430 (46.7) | 301 (52.7) | 154 (48.9) | 361 (48.8) | 0.17 |
| Body mass index, kg/m^2^ | 23.9±3.4 | 23.9±3.3 | 25.1±3.4^*,†^ | 25.0±3.6^*,†^ | <0.001 |
| Current smoker, n (%) | 95 (10.3) | 70 (12.3) | 45 (14.3) | 90 (12.2) | 0.26 |
| Daily drinker, n (%) | 259 (28.2) | 217 (38.0)^*^ | 62 (19.7)^*,†^ | 188 (25.4)^†^ | <0.001 |
| Diabetes mellitus, n (%) | 219 (23.8) | 124 (21.7) | 97 (30.8)^†^ | 200 (27.1) | 0.011 |
| Chronic kidney disease, n (%) | 177 (19.3) | 109 (19.2) | 77 (24.4) | 208 (28.2)^*,†^ | <0.001 |
| Atrial fibrillation, n (%) | 28 (3.0) | 15 (2.6) | 9 (2.9) | 28 (3.8) | 0.65 |
| Sleep apnea syndrome, n (%) | 35 (3.8) | 26 (4.6) | 13 (4.1) | 33 (4.5) | 0.88 |
| Statin use, n (%) | 250 (27.2) | 113 (19.8)^*^ | 81 (25.7) | 161 (21.8) | 0.004 |
| History of CVD, n (%) | 112 (12.2) | 82 (14.4) | 40 (12.7) | 97 (13.1) | 0.68 |
| Fasting glucose, mg/dL | 105.2±24.0 | 105.9±23.1 | 110.4±30.3^*^ | 110.4±31.5^*,†^ | <0.001 |
| Total cholesterol, mg/dL | 204.0±31.5 | 205.5±32.4 | 203.5±32.7 | 204.2±32.4 | 0.78 |
| High-density lipoprotein cholesterol, mg/dL | 58.6±15.1 | 60.6±16.2^*^ | 55.5±15.1^*,†^ | 57.3±15.6^†^ | <0.001 |
| Antihypertensive medication use, n (%) | 740 (80.4) | 488 (85.5) | 261 (82.9) | 612 (82.8) | 0.099 |
| Evening administration, n (%) | 262 (28.5) | 157 (27.5) | 96 (30.5) | 221 (29.9) | 0.71 |
| Number of antihypertensive drugs | 1.6±1.1 | 1.6±1.1 | 1.8±1.3 | 1.7±1.2 | 0.085 |
| Calcium channel blockers, n (%) | 417 (45.3) | 304 (53.2)^*^ | 182 (57.8)^*^ | 403 (54.5)^*^ | <0.001 |
| Angiotensin-converting enzyme inhibitors, n (%) | 45 (4.9) | 36 (6.3) | 31 (9.8)^*^ | 51 (6.9) | 0.018 |
| Angiotensin receptor blockers, n (%) | 484 (52.6) | 277 (48.5) | 162 (51.4) | 395 (53.5) | 0.32 |
| Diuretics, n (%) | 299 (32.5) | 154 (27.0) | 92 (29.2) | 187 (25.3)^*^ | 0.009 |
| β-blockers, n (%) | 129 (14.0) | 89 (15.6) | 58 (18.4) | 115 (15.6) | 0.31 |
| α-blockers, n (%) | 36 (3.9) | 18 (3.2) | 24 (7.6)^*,†^ | 51 (6.9)^*,†^ | 0.001 |
| **BP measures, mmHg** | | | | | |
| Office SBP | 127.0±8.8 | 148.8±9.1^*^ | 130.5±7.0^*,†^ | 153.4±11.9^*,†,‡^ | <0.001 |
| Office DBP | 76.7±7.6 | 86.1±9.3^*^ | 76.5±7.8^†^ | 87.1±10.6^*,‡^ | <0.001 |
| Morning home SBP | 125.8±8.9 | 130.7±8.0^*^ | 146.2±10.5^*,†^ | 150.0±12.7^*,†,‡^ | <0.001 |
| Morning home DBP | 75.1±7.2 | 77.1±8.1^*^ | 82.9±9.3^*,†^ | 84.7±10.1^*,†,‡^ | <0.001 |
| Evening home SBP | 119.3±9.1 | 122.2±8.2^*^ | 139.1±10.1^*,†^ | 141.7±12.1^*,†,‡^ | <0.001 |
| Evening home DBP | 69.1±7.3 | 69.7±7.2 | 77.2±8.6^*,†^ | 78.2±10.1^*,†^ | <0.001 |
| Daytime home SBP | 122.6±7.6 | 126.4±6.1^*^ | 142.6±8.6^*,†^ | 145.8±10.7^*,†,‡^ | <0.001 |
| Daytime home DBP | 72.1±6.6 | 73.4±6.8^*^ | 80.0±8.4^*,†^ | 81.5±9.5^*,†,‡^ | <0.001 |
| Nocturnal home SBP | 113.6±11.4 | 118.1±11.5^*^ | 125.6±12.7^*,†^ | 130.9±14.8^*,†,‡^ | <0.001 |
| Nocturnal home DBP | 66.7±7.0 | 68.7±8.0^*^ | 71.2±8.3^*,†^ | 73.8±9.8^*,†,‡^ | <0.001 |
| Data are expressed as means±SD or number (percentage). To compare characteristics among the groups, we used ANOVA with the Holm correction for multiple comparisons. The daytime home BP values were defined as the average of morning and evening home BP values. Statistical significance was defined as *P*<0.05. ^*^*P*<0.05 vs. daytime normotension group; ^†^*P*<0.05 vs. white-coat daytime hypertension group; ^‡^*P*<0.05 vs. office-masked daytime hypertension group. ANOVA indicates analysis of variance; BP, blood pressure; CVD, cardiovascular disease; DBP, diastolic blood pressure; SBP, systolic blood pressure. | | | | | |

| **Supplemental Table 3. Total cardiovascular events risk of BP phenotypes defined by home BP monitoring in participants using antihypertensive medications** | | | | |
| --- | --- | --- | --- | --- |
| 1. BP phenotypes defined using office BP and **nocturnal home BP** | | | | |
|  | **Nocturnal normotension**  (n=512) | **White-coat**  **nocturnal hypertension**  (n=319) | **Office-masked**  **nocturnal hypertension**  **(**n=489) | **Sustained**  **nocturnal hypertension**  (n=781) |
| Number of events,  (per 1,000 person-years; 95%CIs) | 21, (5.7; 3.8–8.8) | 16, (7.0; 4.3–11.4) | 37, (10.6; 7.7–14.6) | 67, (12.1; 9.5–15.3) |
| Model 1 (unadjusted) | 1.00 [reference] | 1.30 (0.68–2.49) | 1.92 (1.12–3.28) | 2.28 (1.39–3.71) |
| Model 2 (adjusted) | 1.00 [reference] | 1.30 (0.67–2.52) | 1.79 (1.04–3.09) | 2.11 (1.27–3.48) |
| Model 3 (Model 2 + daytime home BP) | 1.00 [reference] | 1.23 (0.63–2.40) | 1.72 (0.98–3.02) | 1.90 (1.09–3.30) |
| 1. BP phenotypes defined using office BP and **daytime home BP** | | | | |
|  | **Daytime normotension**  (n=740) | **White-coat**  **daytime hypertension**  (n=488) | **Office-masked**  **daytime hypertension**  **(**n=261) | **Sustained**  **daytime hypertension**  (n=612) |
| Number of events,  (per 1,000 person-years; 95%CIs) | 40, (7.6; 5.6–10.3) | 30, (8.6; 6.0–12.3) | 18, (9.7; 6.1–15.3) | 53, (12.1; 9.3–15.9) |
| Model 1 (unadjusted) | 1.00 [reference] | 1.23 (0.77–1.98) | 1.46 (0.83–2.54) | 1.77 (1.17–2.67) |
| Model 2 (adjusted) | 1.00 [reference] | 1.19 (0.73–1.92) | 1.13 (0.64–1.99) | 1.55 (1.01–2.37) |
| Model 3 (Model 2 + nocturnal home BP) | 1.00 [reference] | 1.12 (0.69–1.82) | 0.94 (0.52–1.71) | 1.20 (0.75–1.94) |
| The adjusted HR (95%CIs) associated with each BP group is shown. The daytime home BP values were defined as the average of morning and evening home BP values. Adjusted factors for Model 2 included age, gender, body mass index, smoking status, alcohol intake, prevalence of diabetes, prevalence of dyslipidemia, prevalence of chronic kidney disease, history of cardiovascular disease (including angina pectoris, myocardial infarction or stroke), antihypertensive medication use and number of antihypertensive medications. Adjustment factors for Model 3 included Model 2 components and daytime home BP (daytime home SBP + daytime home DBP) or nocturnal home BP (nocturnal home SBP + nocturnal home DBP). BP indicates blood pressure; CI, confidence interval; DBP, diastolic blood pressure; HBP, home blood pressure; HR, hazard ratio; SBP, systolic blood pressure. | | | | |

**Supplemental Figure 1**. The office and home blood pressure monitoring device used in this study (HEM-5001; Medinote, Omron Healthcare, Kyoto, Japan)

**Supplemental Figure 2**. Instruction of home blood pressure measurement

**Supplemental Figure 2 (continued)**

**Supplemental Figure 2 (continued)**

**Supplemental Figure 3**. Flowchart of study participants

**
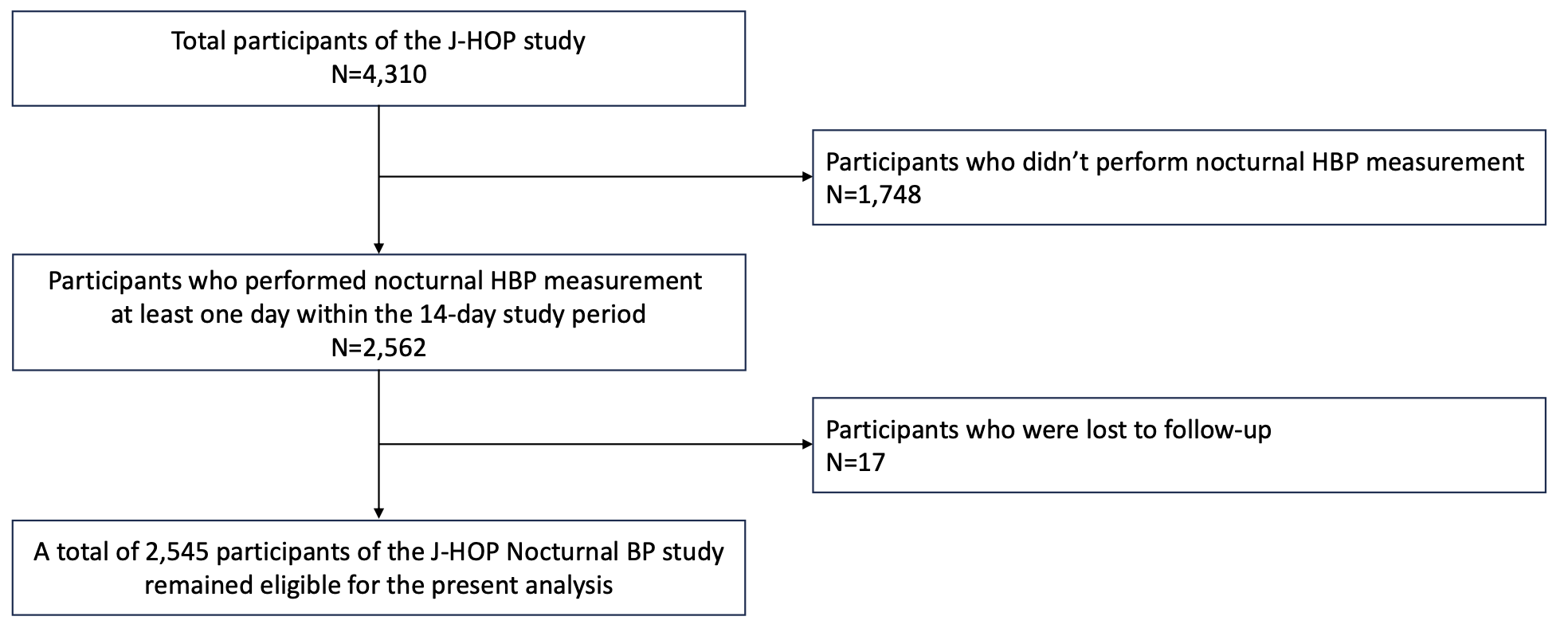
**
